# Consumer-perceived risks and choices about pharmaceuticals in the environment: a cross-sectional study

**DOI:** 10.1186/1476-069X-12-45

**Published:** 2013-06-05

**Authors:** Simone Dohle, Victoria E A Campbell, Joseph L Arvai

**Affiliations:** 1 Institute for Environmental Decisions, ETH Zurich, Zurich, Switzerland; 2 Department of Communication and Culture, University of Calgary, Calgary, Canada; 3 Department of Geography, University of Calgary, Calgary, Canada; 4 Decision Research, Eugene, OR, USA

**Keywords:** Affect, Environment, Health effects, Pharmaceuticals, Risk, Survey

## Abstract

**Background:**

There is increasing concern that pollution from pharmaceuticals used in human medicine and agriculture can be a threat to the environment. Little is known, however, if people are aware that pharmaceuticals may have a detrimental influence on the environment. The present study examines people’s risk perception and choices in regard to environmental risks of pharmaceuticals used in human medicine and for agricultural purposes.

**Methods:**

A representative sample of the U.S. population (N = 640) was surveyed. Respondents completed a hypothetical choice task that involved tradeoffs between human and environmental health. In addition, it was examined how much people would support an environment policy related to drug regulation.

**Results:**

For agricultural pharmaceuticals, respondents reported a high level of satisfaction for a policy requiring farms to limit their use of antibiotics. In the domain of pharmaceuticals used in human medicine, we found that people were willing to consider environmental consequences when choosing a drug, but only when choices were made about treatment options for a rather harmless disease. In contrast, when decisions were made about treatment options for a severe disease, the drug’s effectiveness was the most important criterion.

**Conclusions:**

It can be concluded that the environmental impact of a drug will be hardly considered in decisions about pharmaceuticals for severe diseases like cancer, and this may be due to the fact that these decisions are predominantly affective in nature. However, for less severe health risks, people are willing to balance health and environmental considerations.

## Background

Pharmaceuticals are an indispensable part of life today. A large number of pharmaceuticals are used every year in the treatment, cure, prevention, or diagnosis of diseases or to otherwise enhance people’s physical or mental well being [1]. In addition to their utilization in human medicine, pharmaceuticals are often used for non-therapeutic agricultural purposes. For example, farm animals are routinely fed low doses of antibiotics in order to enhance growth and prevent infection. In fact, over half of the antibiotics that are produced in the U.S. are used in agriculture [2].

In addition to their clear benefits, however, there is growing evidence that pollution from pharmaceuticals can be a severe threat to the environment [3]. For instance, it has been demonstrated that exposure to 17α-ethinyl estradiol (EE2), a steroid hormone used in contraceptives, can cause the feminization of fish [4,5] and amphibians [6]. Serious impacts of pharmaceuticals in the environment are also demonstrated by the dramatic decline of three vulture species in India and Pakistan. The decline of the vulture populations has been connected to diclofenac, a drug that is often used in veterinary medicine and accumulates in treated livestock. Because vultures are feeding not only on carrion of wildlife, but also on domestic livestock and cattle, the residues of diclofenac in livestock were made responsible for the poising and unusually high death rate among the vulture species [7].

There is widespread agreement that many of these negative environmental effects could be avoided through the more careful handling and use of these drugs [8,9]. To this end, the present study aims at assessing people’s awareness of the environmental risk associated with pharmaceuticals used in human medicine and agriculture. More precisely, the main purpose of this research is to examine what kinds of judgments we can expect people to make when they must balance health and environmental considerations.

### Toxicological effects of pharmaceuticals in the environment

A wide range of pharmaceuticals used in human medicine, including anti-inflammatory drugs (e.g., diclofenac), lipid regulators (e.g., bezafibrate) and beta-blockers (e.g., metoprolol) have been found in treated sewage, rivers, seawater, and rarely, in groundwater and drinking water [10,11]. Concentrations of detected compounds are often very low, and therefore do not pose a severe threat for humans or the environment [10,12,13]. However, there are some extreme (but rare) instances where toxicological effects of pharmaceuticals in the environment have serious consequences, such as the examples of the feminization of fish and amphibians mentioned above, and the dramatic decline of vulture species across the Indian subcontinent [4-7].

In addition, there is scientific uncertainty about chronic and mixture effects of pharmaceuticals in the environment [8,13,14]. Investigations of chronic effects, i.e. long-term toxicological consequences, have only rarely been conducted and are therefore only marginally known [for a review, see 11]. Mixture effects refer to the possibility that exposure to certain combinations of pharmaceuticals can result in a risk that is greater than the sum of the risk attributable to each pharmaceutical alone. Occurrence of multiple pharmaceuticals has been reported in the literature [10,13], and thus, health consequences arising from mixtures of pharmaceuticals in the environment are possible [8]. Therefore, because of the scientific uncertainty related to chronic and mixture effects, it seems reasonable to apply the precautionary principle to prevent serious potential harm to humans and animals and to minimize pollution from pharmaceuticals in the environment [12]. In addition, experts agree that more research and further refinement of risk assessment are required to estimate the acute and chronic potential effects of pharmaceuticals in the environment [11,15].

### Emission of pharmaceuticals into the environment

There are different ways in which pharmaceuticals may enter the environment. First, pharmaceuticals used in agriculture enter the environment through the application of manure to fields and subsequent runoff and also through direct application in aqua-culture such as fish farms [11]. Second, pharmaceuticals in human medicine are only partly absorbed after intake, and a significant fraction of the original substance passes through the body unchanged [1,12]. As a result, pharmaceuticals and their metabolites are emitted into raw sewage. Degradation of these substances in the sewage treatment plant depends on the drug types and treatment facilities [8,12]. Some pharmaceuticals are not degraded in sewage treatment plant, resulting in the contamination of rivers, lakes, and even drinking water [1,11]. The disposal of unused or expired pharmaceuticals via the sink or the toilet is a third pathway for emission of pharmaceuticals into the environment [8,12,15,16]. In particular, liquid medicinal products are frequently disposed via the domestic sewage system [8]. Because pharmaceuticals that are disposed via the sink or toilet enter the sewage system in an unmodified form, they may contribute disproportionally to environmental pollution [12].

### Risk management

The fact that pharmaceuticals are regularly flushed from households into the sewage system suggests that there is little awareness about the potential threat of pharmaceuticals to the natural environment [8,12]. In fact, one reason for this method of disposal is linked to concerns that improperly discarded drugs may end up in the hands of children [12]. In addition, many people feel that disposing pharmaceuticals via domestic sewage is unlikely to harm the environment, especially when these pharmaceuticals are “weak” over-the-counter (OTC) drugs. In a study on household disposal of pharmaceuticals, Bound and colleagues [12] found that familiar drugs that are used regularly and available over the counter (e.g., painkillers) are perceived less potent and less threatening to the environment compared to unfamiliar drugs such as antiepileptics. However, painkillers belong to the most frequently detected pharmaceuticals in the aquatic environment [15] and can have severe ecological consequences [17], suggesting that people’s risk perception differs markedly from the actual environmental risks of pharmaceuticals.

Three different approaches for managing risks of pharmaceuticals in the environment can be distinguished: Emission control, drug development, and handling of drugs [8,9]. Emission control (e.g., optimization of treatment options in wastewater management) and drug development (i.e., designing pharmaceuticals that are optimized for both efficacy and degradability) depend largely on scientific research and technological capabilities. In contrast, handling of drugs relates to change in current prescription practices, utilization, and disposal patterns. Environmental labels printed on drug packages, for example, could make a drug’s environmental impact more salient and facilitate decision among physicians, pharmacists, and patients. The Stockholm County Council in Sweden recently developed an environment label for pharmaceuticals, which is aimed at helping health professionals and patients to assess the drug’s potential impact on the environment [18]. The classification system utilizes producer-supplied data on biodegradation, bioaccumulation, and ecotoxicity. Each of the variables is assigned a value between zero and three, and the sum of these values yields an index that varies between zero and nine. Where medications of similar action and efficiency are available, the classification system is intended to offer a simple but accurate tool to select the most environmentally friendly option. It is unclear, however, if people would take into account the environmental impact of a pharmaceutical when choosing between different drugs.

### Severity of illness

When people must decide between different drugs or treatment options, many factors need to be considered, and it is likely that the decision to treat an illness will only be relevant when people perceive a significant or threatening change in their health status (ranging from disruptive but temporary illnesses to severe or life threatening conditions).

Research in the area of social cognition, for example, links patients’ and consumers’ decisions about treatments (or behaviour change) to perceptions of health risk. In this regard, the Health Belief Model [19] posits that (i) perceptions regarding the risk of contracting an illness, (ii) the severity of an illness or its symptoms, (iii) perceived barriers to changes in behavior that may reduce risk or severity, and the (iv) perceived benefits of interventions or treatments are all key factors that influence decisions about treatments intended to ameliorate health risk. In a similar vein, Turk and colleagues [20] suggested that the judged severity of an illness (i.e., the individual's knowledge about the degree to which a disease is contagious, difficult to cure, and chronic) could be one major factor that may influence people’s treatment decisions and coping responses.

The important role of risk perceptions as well as the severity of illnesses in treatment decisions was also highlighted in recent studies about the acceptance of generic drugs. These studies examined whether consumers were more likely to accept generic or branded drugs for harmless or more severe illnesses. It was revealed that many consumers accept generic drugs for the treatment of relatively harmless or benign illnesses like flu. In contrast, for severe health problems like asthma or angina pectoris, consumers were less likely to accept generic drugs, presumably because they feared that generic drugs could be ineffective [21-23]. Therefore, the severity of the illness is an important factor that needs to be considered in studies about consumer-perceived risks and decisions about drugs.

### Objectives of the present study

The aim of the present study was threefold. First, we sought to explore if people are aware that pharmaceuticals used in human medicine or for agricultural purposes can be a threat to the environment. In particular, we asked people to compare the threat of pharmaceuticals in the environment to other (well-known) threats to the environment. A second aim was to investigate how satisfied people would be with an environmentally friendly pharmaceutical if this specific drug were the only one that was available to them. Third, the objective of the present study was to examine if environment protection plays a role in people’s drug choices.

In line with other studies [21,22], we anticipated that the severity of an illness has important consequences for drug choices: We hypothesized that the environmental impact of a pharmaceutical used in human medicine would only be considered when the pharmaceutical was used for a rather harmless or benign ailment. Two different scenarios in human medicine were compared: One scenario depicted a severe health risk (cancer), while the other scenario depicted a more benign health risk (common cold). For both scenarios, people had to make trade-offs between effectiveness and environmental protection.

In addition, we also examined decisions about agricultural pharmaceuticals in a third scenario to examine how these choices may differ from those made about pharmaceuticals used in human medicine. These three scenarios allowed us to compare risk perceptions and drug choice in situations where the consequences of disease are of direct relevance to humans (i.e., cancer and the common cold) or only indirect relevance (i.e., preventing disease in food animals). We hypothesized that people would be more willing to address the environmental consequences of drugs used in agricultural production since the benefits to humans were indirect. Moreover, this paper examined how much people would support an environment policy related to drug regulation. Specifically, we wanted to explore the acceptance of certain management strategies. We were interested in determining if people would support a law requiring physicians to prescribe the drug with the lowest environmental impact, and if they would accept a label describing the negative environmental impact of OTC drugs.

In order to allow for inferences to be drawn about the generalizability of our findings, we surveyed a representative sample of the United States (U.S.) population. Compared to other countries, there is a large market for OTC drugs in the U.S. coupled with strong libertarian tendencies amongst its citizens; taken together, this suggests that U.S. residents are quite familiar with choosing among different pharmaceutical options. More than 80 therapeutic categories of OTC drugs exist currently in the United States, ranging from external and internal analgesics to sunscreens, weight control drug products, and other topical products with a therapeutic effect [24]. Two different age groups were sampled, because decisions in regard to health are often markedly different between older people and younger people [e.g., [25].

## Methods

### Sampling and data collection

We used a pre-recruited web-enabled panel developed by Knowledge Networks (KN). Each participant was randomly drawn from a probability sample of active members in KN’s database. The sampling approach for this study was designed to increase the demographic similarities between those who completed the study protocol and U.S. Federal Census benchmarks by factoring demographic groups’ estimated response rates into the initial sample draw. The probability that members of a given demographic group would take part in the study was factored into the overall sampling frame. Consequently, the sampling frame consisted of oversampled groups that tend to have a lower response and consistency rate and undersampled groups that tend to have a higher response and consistency rate. As a result, the set of completed questionnaires is representative of and closely mirrors the U.S. population on key demographic characteristics [26].

### Participants

The sample for the survey consisted of adults in the general population within two age groups (20–39; 40–60). Six hundred forty (N = 640) people participated in the survey (males: n = 314; 49%). Fifty per cent (n = 317) were between the ages of 20 and 39, and 50% were between 40 and 60 (n = 323). The mean survey completion rate was 51%. The survey completion rate was lower for the younger age group (42%) than for the older age group (65%).

### Questionnaire

#### Risk perceptions

In the first part of the questionnaire, participants evaluated each of 20 different risks to the natural environment. Perception for every risk to the natural environment was indicated on a scale from 1 (“not at all severe”) to 10 (“very severe)”; the values 5 and 6 corresponded to “moderately severe”. For a complete list of the risks that were considered, please refer to Table 1.

**Table 1:** Means and standard deviations of perception of risks to the natural environment

	**All**	**Gender**			**Age groups**		
		**Females**	**Males**	**p**	**20**-**39**	**40**-**60**	**p**
CO_2_ emissions from industry	7.06 (2.34)	7.44 (2.14)	6.67 (2.46)	<.001^*^	6.95 (2.28)	7.17 (2.39)	.234
CO_2_ emissions from automobiles	6.78 (2.33)	7.09 (2.16)	6.46 (2.46)	.001^*^	6.67 (2.25)	6.89 (2.41)	.224
Garbage and landfills	6.70 (2.27)	7.02 (2.15)	6.37 (2.34)	<.001^*^	6.61 (2.14)	6.79 (2.39)	.314
The growing size of the human population	6.64 (2.45)	6.64 (2.43)	6.65 (2.47)	.925	6.51 (2.48)	6.78 (2.42)	.172
Large-scale logging and forestry operations	6.36 (2.47)	6.57 (2.38)	6.14 (2.54)	.032	6.19 (2.43)	6.52 (2.50)	.099
Suburban development and sprawl	6.24 (2.26)	6.41 (2.26)	6.06 (2.26)	.055	6.07 (2.24)	6.40 (2.27)	.069
**Pharmaceuticals (drugs) used in agriculture**	6.15 (2.39)	6.49 (2.35)	5.79 (2.37)	<.001^*^	5.85 (2.25)	6.44 (2.48)	.002^*^
Overfishing	6.11 (2.41)	6.00 (2.46)	6.23 (2.37)	.234	5.92 (2.31)	6.30 (2.50)	.051
Illegal hunting and poaching	6.01 (2.56)	6.32 (2.50)	5.70 (2.58)	.002^*^	5.81 (2.42)	6.21 (2.67)	.050
Coal-fired power plant	6.00 (2.40)	6.00 (2.43)	6.01 (2.36)	.960	5.92 (2.36)	6.08 (2.44)	.398
Invasive species	5.95 (2.39)	6.06 (2.39)	5.84 (2.38)	.259	5.59 (2.34)	6.30 (2.38)	<.001^*^
Nuclear power	5.89 (2.72)	6.31 (2.65)	5.47 (2.71)	<.001^*^	5.75 (2.52)	6.03 (2.89)	.186
Oil and gas exploration	5.65 (2.69)	5.90 (2.64)	5.39 (2.72)	.016	5.78 (2.60)	5.52 (2.77)	.229
Genetic engineering	5.53 (2.57)	5.87 (2.56)	5.17 (2.53)	.001^*^	5.29 (2.46)	5.76 (2.65)	.022
Mining	5.51 (2.28)	5.60 (2.28)	5.41 (2.27)	.286	5.40 (2.21)	5.61 (2.34)	.248
**Pharmaceuticals (drugs) used in human medicine**	5.34 (2.50)	5.55 (2.44)	5.12 (2.54)	.030	5.00 (2.33)	5.66 (2.61)	.001^*^
Large-scale farming operations	4.92 (2.30)	4.96 (2.29)	4.88 (2.30)	.658	4.80 (2.12)	5.03 (2.46)	.203
Nanotechnology	4.45 (2.18)	4.67 (2.18)	4.23 (2.15)	.011	4.27 (1.98)	4.63 (2.35)	.038
Hydroelectric dams	4.14 (2.23)	4.31 (2.24)	3.96 (2.22)	.048	3.96 (2.03)	4.31 (2.40)	.052
Wind power (windmills)	2.76 (2.21)	2.73 (2.11)	2.79 (2.31)	.741	2.72 (2.09)	2.80 (2.32)	.650

Note. ^*^ Significant at Bonferroni corrected level of significance, 0.0025 (0.05/20). Data are sorted in descending order of mean risk ratings. Standard deviations are given in parentheses.

#### Pharmaceuticals in human medicine

In the next part of the questionnaire, participants were confronted with two scenarios related to pharmaceuticals used in human medicine. The first scenario dealt with hypothetical pharmaceutical drugs used for the treatment of cancer, while the second scenario described hypothetical pharmaceuticals for the common cold. In both cases, we created realistic but hypothetical names for both the drugs, and—in the case of cancer—the ailment, so as to avoid recognition bias on the part of respondents. Although we cannot exclude that some hypothetical names may evoke unintended connotations in some participants, we suggest that such connotations would be much stronger in the case of real names.

Scenario 1. In the first scenario, participants were informed that:

Waverly’s Carcinoma is a form of skin cancer in humans that, if left untreated, can lead to death. However, if this form of cancer is detected at an early stage and treated with a specific drug, a majority of people can be cured allowing them to lead long and productive lives that are free of this disease.

While effective, many of the newer drugs used to treat Waverly’s Carcinoma are potentially harmful to the natural environment. For example, after being consumed and then excreted by humans, some of these drugs make their way into rivers and lakes where they have harmful effects on many species of fish. Rainbow trout are particularly sensitive to these drugs; even minor exposure to these drugs results in a sharp decline in the reproductive rate of these fish.

Participants were asked to assume that they have just been diagnosed with the very early stages of Waverly’s Carcinoma. Their doctor would give them a choice between two drugs to treat the disease. Then, participants were confronted with a table that showed the relative age, effectiveness, and negative environmental consequences of the two drugs.

The experiment was counterbalanced with each individual receiving only one version (of three) of a table that described two drugs. In the first version, participants compared a drug called Alocyl (Option 1) to a drug called Botorex (Option 2). In the second version, participants compared Alocyl (Option 1) to Cetozol (Option 2), and in the third version, Botorex (Option 1) to Cetozol (Option 2). Table 2 shows all three pharmaceuticals and indicates how they differ in regard to relative age, effectiveness, and negative environmental consequences. Importantly, in each of the three versions of the experiment, Option 1 was always newer and was also more effective than Option 2. Option 2, in contrast, was always less harmful to the environment than Option 1.

**Table 2:** **Human medicine**: **pharmaceuticals for cancer**

	**Alocyl**	**Botorex**	**Cetozol**
Relative age of drug	Newer	Older (Newer)	Older
Per cent of patients that are cancer-free (i.e., cured) following treatment	90%	70%	50%
Per cent decline in the reproductive rate of Rainbow Trout	40%	20%	0%

Note. Botorex was older when compared to Alocyl, but newer when compared to Cetozol.

After reading the table, participants were asked four questions. First, they were asked about satisfaction with Option 1; subsequently, they were asked about satisfaction with Option 2 (“Assume that Option 1 [Option 2] was the only drug available to you. Under these circumstances, how satisfied would you be with this drug considering all of its characteristics (its relative age, effectiveness, and environmental impact)?”). Preferences were expressed on a 1–10 scale (where 1 = “not at all satisfied”, 5 and 6 = “moderately satisfied”, and 10 = “extremely satisfied”).

The third question was a choice task between Option 1 and 2 (“Assuming you could select between both drugs, which would you choose?”). Participants could choose one of the two options, or could indicate that they would opt out of treatment, i.e., that they would refuse to take either drug.

The fourth question was related to the environmental impact of the drug (“In some countries, doctors may be required by law to prescribe the drug with the lowest environmental impact; how would you feel if this was the case in the United States?”). Preferences for this policy were expressed on a 1–10 scale (where 1 = “I would be strongly opposed to this policy”, 5 and 6 = “I would be indifferent to this policy”, and 10 = “I would strongly support this policy”).

Scenario 2. The second scenario was about the common cold. Participants were provided with the following information:

The virus that causes the common cold is not lethal and makes people feel sick for approximately 72 hours (3 full days). The common symptoms include a runny nose, a burning feeling in the nose or throat, a mild cough, and the general feeling of being tired and unwell.

There are several classes of drugs that can be used to treat cold symptoms. Newer drugs are generally more effective at alleviating these symptoms while older ones are less effective. However, effectiveness comes with a price. The newer more effective drugs are potentially harmful to the natural environment. For example, traces of some drugs have been found in the eggshells of many common birds, which results in declines in the populations of affected species.

Participants were asked to assume that they have just caught a cold. The pharmacist at their local drugstore suggests two drugs that treat cold symptoms. Like in Scenario 1, participants were confronted with a table that showed the relative age, effectiveness, and negative environmental consequences of the two drugs (see Table 3).

**Table 3:** **Human medicine**: **pharmaceuticals for the common cold**

	**Fluvex**	**Getaway**	**Hydramax**
Relative age of drug	Newer	Older (Newer)	Older
Per cent of patients that are symptom-free after 24 hours	90%	70%	50%
Per cent decline in affected bird populations	40%	20%	0%

Note. Getaway was older when compared to Fluvex, but newer when compared to Hydramax.

Again, the experiment was counterbalanced, and each individual received only one version (of three) of a table comparing two drugs. In a first version, participants compared a drug named Fluvex (Option 1) to a drug named Getaway (Option 2). The second version contrasted Fluvex (Option 1) with Hydramax (Option 2), and the third version Getaway (Option 1) with Hydramax (Option 2). As above, Option 1 was always newer and more effective, while Option 2 was always less harmful to the environment.

The same questions as in Scenario 1 were asked, i.e. (a) satisfaction with Option 1, (b) satisfaction with Option 2, (c) choice between Option 1 and 2, and (d) support of environment policy. Support of environment policy was queried in a slightly different way in the second scenario, given the fact that cold drugs that treat cold symptoms are normally sold over the counter. Hence, the reading of the last question was: “In some countries, over-the-counter drugs are required by law to carry a label describing their negative environmental impacts. How would you feel if this was the case in the United States?”.

#### Pharmaceuticals in agriculture

Scenario 3. A third scenario was about antibiotics used in agriculture. Participants read the following description:

Antibiotics are used widely during the raising of animals (e.g., beef, pork, chickens, etc.) in agriculture. Rather than using these antibiotics to treat a specific, current infection (as in human medicine), antibiotics in agriculture are typically added to animal feed as a means of preventing future infections in the animals and enhancing their growth.

Using antibiotics as a preventative medicine has potential problems. Chief among these is that antibiotic use in agriculture contributes to the development of antibiotic-resistant bacteria in nature. These bacteria are often referred to as “superbugs” because they cannot be treated with conventional drugs. These superbugs may, in turn, infect both humans and other animals.

Participants were then asked to assume that a ballot initiative asked for their opinion about the use of antibiotics in agriculture, specifically related to raising beef cattle. Two options for preventing infections in cattle were presented (see Table 4). Three different versions were used in the experiment. In all three versions, Option 1 was more effective (i.e., indicted a higher percept of cattle that were free of infection over their lifetime). Option 2, in contrast, was always characterized by a smaller number of antibiotic resistant bacteria; thus, it was more environmentally friendly option.

**Table 4:** **Agriculture**: **antibiotics in animal feed**

	**No change to current regulations surrounding the use of antibiotics**	**A 50% reduction in the amount of antibiotics that may be used**	**A ban on the use of antibiotics in animal feed**
Per cent of farmed cattle that are free of infection over their lifetime	90%	70%	50%
Per cent increase in the number of antibiotic resistant bacteria	40%	20%	0%

Subsequently, participants were asked about (a) satisfaction with Option 1, (b) satisfaction with Option 2, (c) choice between Option 1 and 2, and (d) support of environment policy. The last question, support for an environmental policy, was related to a limitation of antibiotics (“In some countries, farms are required by law to limit their use of antibiotics in animal feed; how would you feel if this was the case in the United States?”) and could be answered on a 1–10 scale (where 1 = “I would be strongly opposed to this policy”, 5 and 6 = “I would be indifferent to this policy”, and 10 = “I would strongly support this policy”).

#### Importance of different food characteristics

Finally, participants were presented a list of 12 different characteristics that food producers might emphasize when marketing their products. For each characteristic in the list, participants indicated how important it is to them when they make decisions about the food they buy at the grocery store. “Antibiotic and/or hormone free” was one of the characteristics (for a complete list of the food characteristics, see Table 5). Importance was expressed on a 1–10 scale (where 1 = “not at all important”, 5 and 6 = “average”, and 10 = “very important”).

**Table 5:** Means and standard deviations regarding importance of food characteristics

	**All**	**Gender**			**Age groups**		
		**Females**	**Males**	**p**	**20**-**39**	**40**-**60**	**p**
Taste/Flavor	8.35 (1.83)	8.51 (1.74)	8.18 (1.91)	.026	8.19 (1.80)	8.50 (1.86)	.037
Price	7.93 (2.01)	8.10 (1.89)	7.75 (2.12)	.031	7.87 (1.96)	7.99 (2.06)	.479
Pesticides and/or herbicide free	7.27 (2.33)	7.63 (2.20)	6.90 (2.41)	<.001^*^	7.02 (2.36)	7.51 (2.28)	.009
Antibiotic and/or hormone free	7.04 (2.41)	7.35 (2.31)	6.73 (2.47)	.001^*^	6.81 (2.45)	7.27 (2.35)	.020
Country and/or U.S. State of origin	6.90 (2.38)	7.18 (2.31)	6.62 (2.41)	.003^*^	6.55 (2.31)	7.24 (2.39)	<.001^*^
Locally grown	6.88 (2.28)	7.17 (2.20)	6.59 (2.32)	.001^*^	6.54 (2.30)	7.22 (2.21)	<.001^*^
Convenience (easy to handle and widely available)	6.83 (2.08)	6.94 (2.13)	6.71 (2.03)	.156	6.76 (1.99)	6.89 (2.17)	.426
In-season	6.75 (2.26)	7.03 (2.13)	6.46 (2.35)	.002^*^	6.47 (2.28)	7.02 (2.20)	.003^*^
Fair wages for farm workers	6.52 (2.42)	6.71 (2.30)	6.32 (2.52)	.042	6.20 (2.34)	6.82 (2.45)	.001^*^
Animal welfare certification	6.13 (2.58)	6.60 (2.43)	5.64 (2.64)	<.001^*^	5.91 (2.42)	6.34 (2.71)	.039
Contribution to climate change	5.71 (2.46)	6.03 (2.34)	5.38 (2.54)	.001^*^	5.47 (2.39)	5.95 (2.50)	.014
Organic certification	5.59 (2.66)	5.94 (2.68)	5.23 (2.60)	.001^*^	5.49 (2.53)	5.69 (2.78)	.357

Note. ^*^ Significant at Bonferroni corrected level of significance, 0.0042 (0.05/12). Data are sorted in descending order of mean importance ratings. Standard deviations are given in parentheses.

## Results

### Risk perceptions

Results indicated that CO_2_ emissions from industry and from automobiles were perceived as the most severe risks to the natural environment. In contrast, wind power was perceived as least severe. Regarding pharmaceuticals, those drugs that were used in agriculture (M = 6.15, SD = 2.39) were perceived as more severe that those used in human medicine (M = 5.34, SD = 2.50). A paired sample t-test showed that this difference was significant, t(626) = 9.77, p < .001.

In addition, male participants evaluated some risks as less severe than female participants (see Table 1). In particular, CO_2_ emissions from industry and from automobiles, garbage and landfills, pharmaceuticals used in agriculture, illegal hunting and poaching, nuclear power, and genetic engineering were perceived as less severe by males. We also found that risk perception differed between the two age groups. Compared to younger participants (age group: 20–39), older participants (age group: 40–60) perceived invasive species and pharmaceuticals used in agriculture and human medicine as more severe.

### Pharmaceuticals in human medicine

#### Satisfaction with option 1

In both the cancer and the common cold scenario, participants were asked how satisfied they would be with Option 1, given this drug was the only drug available to them. In all three versions of the cancer scenario, individuals were satisfied with Option 1, i.e. the drug that was more effective but less environmentally friendly (Version 1: M = 6.56, SD = 2.45; Version 2: M = 6.29, SD = 2.54; Version 3: M = 6.32, SD = 2.28). In contrast, in the common cold scenario, individuals were less satisfied with Option 1 (Version 1: M = 4.54, SD = 2.71; Version 2: M = 4.47, SD = 2.88; Version 3: M = 4.57, SD = 2.70). To test whether satisfaction with Option 1 was significantly different between the two scenarios, we pooled the three versions of the experiment. A mixed ANOVA with Scenario (cancer vs. common cold) as within-subject factor and Age-groups (20–39 vs. 40–60) and Gender (males vs. females) as between-subject factors indicated that the difference was significant, F (1, 622) = 315.32, p < .001. Participants were more satisfied with the effective but less environmentally friendly drug (Option 1) in the cancer scenario compared to the common cold scenario (see Table 6). Satisfaction with Option 1 was also influenced by age, F(1, 622) = 20.68, p < .001, and gender, F(1, 622) = 5.98, p = .015. In general, younger participants (age group 20–39) and males were more satisfied with Option 1, thus, effectiveness was more important for them. None of the interactions between Scenario and Age-groups or Gender were significant; all p > .05.

**Table 6:** **Means and standard deviations of satisfaction with Option 1 and Option 2**, **and support for environmental policy**

-			**Option 1**		**Option 2**		**Support for policy**	
	**Gender**	**Age groups**	**M**	**SD**	**M**	**SD**	**M**	**SD**
Human Medicine: Cancer Scenario	Males	Age: 20-39	6.72	2.15	6.06	2.03	4.24	2.56
		Age: 40-60	6.50	2.47	6.10	2.09	4.62	3.02
		Total	6.61	2.32	6.08	2.06	4.43	2.80
	Females	Age: 20-39	6.71	2.13	6.02	1.89	4.94	2.79
		Age: 40-60	5.77	2.74	5.68	2.17	4.61	2.86
		Total	6.23	2.50	5.85	2.04	4.78	2.82
	Total	Age: 20-39	6.72	2.14	6.04	1.96	4.60	2.70
		Age: 40-60	6.13	2.63	5.89	2.14	4.62	2.93
		Total	6.42	2.42	5.96	2.05	4.61	2.82
Human Medicine: Common Cold Scenario	Males	Age: 20-39	5.10	2.77	6.10	2.34	6.91	2.54
		Age: 40-60	4.42	2.59	6.08	2.68	6.99	2.65
		Total	4.75	2.70	6.09	2.51	6.95	2.59
	Females	Age: 20-39	4.94	2.77	6.22	2.28	7.55	2.34
		Age: 40-60	3.63	2.63	5.61	2.85	7.52	2.65
		Total	4.28	2.78	5.91	2.60	7.54	2.50
	Total	Age: 20-39	5.02	2.77	6.16	2.31	7.24	2.45
		Age: 40-60	4.02	2.64	5.84	2.77	7.26	2.66
		Total	4.51	2.75	6.00	2.56	7.25	2.56
Agriculture: Antibiotics	Males	Age: 20-39	4.51	2.63	6.42	2.14	6.72	2.47
		Age: 40-60	4.19	2.51	6.41	2.54	6.68	2.69
		Total	4.35	2.57	6.41	2.35	6.70	2.58
	Females	Age: 20-39	4.42	2.33	6.44	2.13	7.52	2.19
		Age: 40-60	3.39	2.29	6.30	2.58	7.61	2.33
		Total	3.90	2.37	6.37	2.37	7.57	2.26
	Total	Age: 20-39	4.47	2.48	6.43	2.13	7.13	2.36
		Age: 40-60	3.78	2.43	6.35	2.56	7.15	2.55
		Total	4.12	2.48	6.39	2.36	7.14	2.46

#### Satisfaction with option 2

Participants were also asked about satisfaction with Option 2, i.e. the drug that that was more environmentally friendly but less effective. For the three versions of the cancer scenario, participants were moderately satisfied with the environmentally friendly Option 2 (Version 1: M = 5.99, SD = 2.04; Version 2: M = 5.68, SD = 2.12; Version 3: M = 6.20, SD = 1.98). A similar picture emerged for the common cold scenario (Version 1: M = 5.27, SD = 2.38; Version 2: M = 6.35, SD = 2.58; Version 3: M = 6.44, SD = 2.56). Again, the three versions of the experiment were pooled to test whether satisfaction with Option 2 was significantly different between the two scenarios. A mixed ANOVA with Scenario (cancer vs. common cold) as within-subject factor and Age-groups (20–39 vs. 40–60) and Gender (males vs. females) as between-subject factors showed no difference in satisfaction with Option 2, neither for the factor Scenario nor for the factors Age-groups and Gender or their interactions, all p > .12. Thus, regardless of age, gender or illness, participants indicated that they would be moderately satisfied with the environmentally friendly drug if this were the only drug available to them (see Table 6).

#### Choice between option 1 and 2

**Figure 1:**
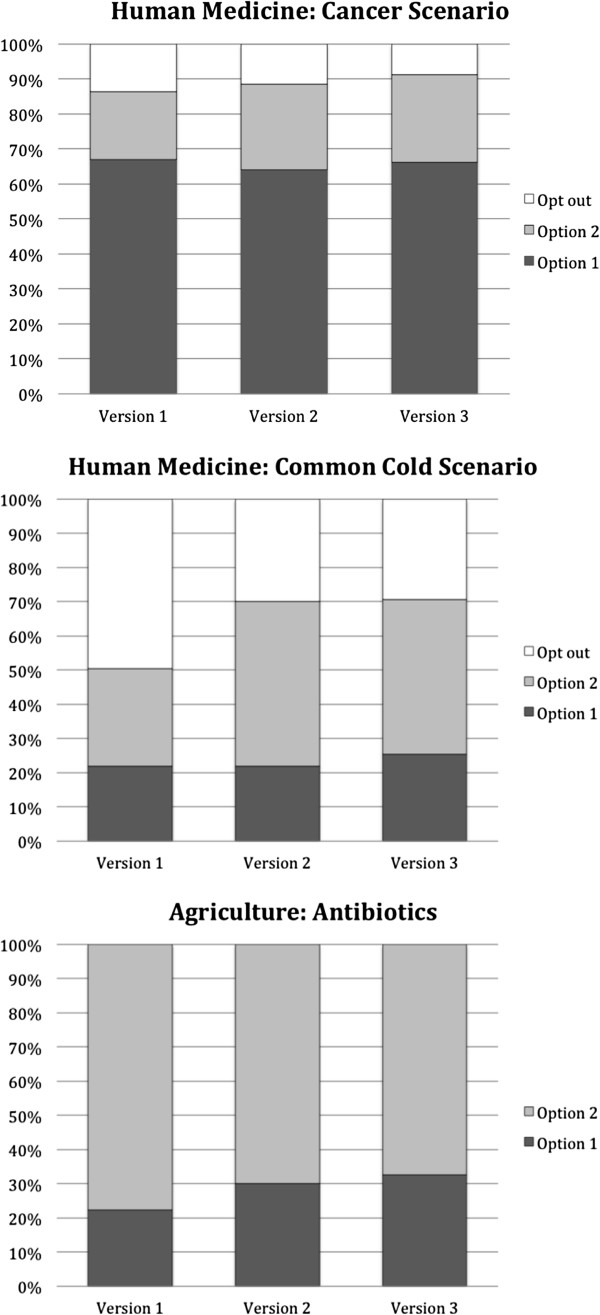
**Results of choice task for the scenarios related to human medicine (cancer and common cold**) **and agriculture (antibiotics) for all three versions of the scenarios.** Versions 1–3 represent comparisons between pairs of drugs that varied in their relative effectiveness and environmental impact (see Tables 2 and 3). Note that Option 1 was always more effective, while Option 2 was always more environmentally friendly.

Results of the choice task were markedly different between the cancer and the common cold scenarios (see Figure 1). Different drug characteristics served as a basis for deciding between the drugs. In the cancer scenario, participants were more likely to choose Option 1, which was always the most effective drug (Version 1, Alocyl vs. Botorex: χ^2^ (2) = 121.25, p < .001; Version 2, Alocyl vs. Cetozol: χ^2^ (2) = 86.47, p < .001; Version 3, Botorex vs. Cetozol: χ^2^ (2) = 107.03, p < .001). In contrast, in the common cold scenario, more participants opted out of treatment when both drugs had a negative environmental impact (Version 1, Fluvex vs. Getaway: χ^2^ (2) = 28.03, p < .001), or they chose the drug with the lowest environmental impact (Version 2, Fluvex vs. Hydramax: χ^2^ (2) = 21.28, p < .001; Version 3, Getaway vs. Hydramax: χ^2^ (2) = 13.63, p = .001).

#### Support of environment policy

Participants were also asked about their support for a policy aimed at lowering the negative environmental impact of drugs. In the cancer scenario, the environmental policy requires doctors to prescribe the drug with the lowest environmental impact; in the common cold scenario, the policy is based on label information. However, because the suggested policies in the two human medicine scenarios were different, direct comparisons between the two policies should be interpreted with care. In the cancer scenario, participants were rather opposed to a policy requiring doctors to prescribe the drug with the lowest environmental impact (Version 1: M = 4.70, SD = 2.81; Version 2: M = 4.33, SD = 2.78; Version 3: M = 4.76, SD = 2.85). In the common cold scenario, participants were supportive of a label describing the negative environmental impact of OTC drugs (Version 1: M = 7.21, SD = 2.53; Version 2: M = 7.37, SD = 2.53; Version 3: M = 7.17, SD = 2.61).

A mixed ANOVA with Scenario (cancer vs. common cold) as within-subject factor and Age-groups (20–39 vs. 40–60) and Gender (males vs. females) as between-subject factors disclosed that support for the policy differed significantly between scenarios, F(1, 627) = 527.97, p < .001. Participants more strongly supported a policy that regulates labelling of OTC drugs (common cold scenario) than a policy that requires doctors to prescribe drugs with the lowest environmental impact (cancer scenario; see Table 6 for means and standard deviations). There was also a main effect for Gender, F(1, 627) = 6.69, p = .010, indicating that females were generally more in favor of both of these two policies. No main effect for Age-groups or interactions between Scenario and Age-groups or Gender was found, all *p* > .19.

### Pharmaceuticals in agriculture

#### Satisfaction with option 1

In all three versions of the scenario about antibiotics used in agriculture, individuals were rather unsatisfied with Option 1, i.e. the drug that was more effective but less environmentally friendly (Version 1: M = 3.83, SD = 2.37; Version 2: M = 4.11, SD = 2.56; Version 3: M = 4.43, SD = 2.48). We pooled the three versions of the experiment to analyze if satisfaction with Option 1 differed between age-groups and gender. A two-way independent ANOVA indicated a main effect for both Age-groups, F(1, 630) = 12.27, p < .001, and Gender, F(1, 630) = 5.32, p = .021, but no interaction between these two variables, p > .05. Older participants (age group 40–60) and females were less satisfied with Option 1; thus, they cared more about antibiotic resistant bacteria and less about the number of cattle that would be free of infection over their lifetime.

#### Satisfaction with option 2

Participants indicated that they would be satisfied, if Option 2 was the only alternative (Version 1: M = 5.88, SD = 2.40; Version 2: M = 6.56, SD = 2.36; Version 3: M = 6.72, SD = 2.24). A two-way independent ANOVA with Age-groups and Gender as between-subject factors yielded no significant main or interaction effect on satisfaction (pooled), all p > .68.

#### Choice between option 1 and 2

If both options were available, participants were more likely to choose Option 2, which was always the most environmentally friendly option (Version 1: χ^2^ (1) = 63.09, p < .001; Version 2: χ^2^ (1) = 33.92, p < .001; Version 3: χ^2^ (1) = 24.92, p < .001). Thus, faced with a trade-off between antibiotic resistant bacteria and the number of infection-free cattle, participants cared more about the consequences of antibiotic resistant bacteria.

#### Support of environment policy

Generally, participants supported a law requiring farms to limit their use of antibiotics in animal feed (Version 1: M = 7.28, SD = 2.43; Version 2: M = 7.00, SD = 2.41; Version 3: M = 7.14, SD = 2.54). A two-way independent ANOVA with Age-groups and Gender as between-subject factors indicated that support for this law was even higher among females, F(1, 628) = 20.12, p < .001. No main effect for age or interaction effect between age and gender was found, both p > .75 (see also Table 6).

#### Importance of food characteristics

Table 5 indicates how important different food characteristics were to participants. When they made decisions about the food they buy at the grocery store, taste and price were the top-ranking characteristics for all participants. In contrast, the food characteristic “Antibiotic and/or hormone free” was ranked fourth. This characteristic was significantly more important for females than for males (see Table 5), but there was no difference between the two age-groups.

## Discussion

There has been an increasing concern that pollution from pharmaceuticals used in human medicine and agriculture can be a threat to the environment [1,3,4,7,8,11-13,15,18]. Little is known, however, about people’s perception of the environmental risk of pharmaceuticals.

In the present study, consumers’ evaluations of various risks to the natural environment indicated that people seem to be aware of the risks associated with the use of pharmaceutical products in agriculture. In contrast, drugs used in human medicine were considered as a less severe problem (Table 1). This is likely related to the fact that respondents see more benefits related to pharmaceuticals used in human medicine. It is well established that risk perception is strongly connected to benefit perception [27,28]. Both benefits and risks are closely linked to affective responses (i.e., the specific quality of “goodness” or “badness”, which is experienced as a feeling state and which demarcates a positive or negative quality of a stimulus), specifically arousal in our case. Using affective responses to stimuli as a mental shortcut is referred to as the affect heuristic [27-30].

Results of the present study suggest that people also use an affect heuristic [29] when they must balance health and environmental considerations. Two scenarios relevant for human health were compared: One scenario dealt with treatment decisions related to a very severe health risk, while the other scenario illustrated a rather benign health risk. The results indicated that people seem to be willing to consider environmental consequences when making decisions about pharmaceuticals for a harmless, but not for a severe disease. When the decision is about life and death, effectiveness is of vital importance. We suspect—in line with previous research [31]—that the cancer scenario elicited a strongly negative affective response, e.g., a strong feeling of dread, which guided the decision and choices between the pharmaceuticals. It should be noted, however, that affect was not measured directly in this study, and further studies should provide more direct evidence that supports this hypothesis.

Our results have consequences for pharmaceutical risk management. In cases where the drug is used for the treatment of a rather harmless disease, people value environmentally friendly options or prefer to refrain from taking medication at all when they realize that the drug has a considerable environmental impact. For OTC drugs, people also tended to be supportive of labels that aimed at recognizing environmentally friendly drugs. However, in the cancer scenario, it was demonstrated that a policy requiring doctors to prescribe the drug with the lowest environmental impact was not supported and probably perceived as too paternalistic.

When weighting human health and environment, people seem to feel that the patient’s health should be considered first, which is also in line with the general ethical standards of physicians [cf. [32]. It should be noted that the environment label developed by the Swedish Stockholm County Council is intended for treatment decisions only when drugs of similar action and efficiency are available in order to select the most environmentally friendly treatment [1,18]. Thus, in one respect almost all the stakeholders are unanimous: Priority should be given to the best possible strategy for curing or palliation of a disease [9].

Concerning pharmaceuticals used in agriculture, respondents generally preferred the more environmentally friendly options. They also reported a high level of satisfaction for a policy requiring farms to limit their use of antibiotics. However, when we asked people about the characteristics they take into account when they buy food in a grocery store, taste and price were the most important drivers for food choices. It must be stressed that abandoning or limiting antibiotics in agriculture may lead to higher prices for some foods, because antibiotics used for growth promotion affect production efficiency and profitability [33]. Hence, limiting the use of antibiotics in agriculture is partly in contradiction to people’s preferences regarding food choices. Risk communicators as well as risk managers are therefore well advised to inform the public that policies aimed at abandoning or limiting antibiotics in agriculture may not be possible without a rise in food prices.

We suspect that the same may be true of OTC drugs, where price signals may interact with other variables in terms of consumer choices. Specifically, more expensive OTC drugs may cause consumers to downplay environmental considerations when making choices about which products to buy. However, in the case of pharmaceuticals for the most serious of ailments, we suspect that price would not be a key driver of choice; here we might expect effectiveness of the drug to be the key driver of choice. Each of these possible interactions should, in our view, be the focus of subsequent research.

We also found considerable differences in risk perception and choices between the two age groups. Compared to older respondents, younger participants seem to be less aware that pharmaceuticals used in human medicine and agriculture can be a threat to the environment. In addition, younger adults (and males) also seem to rely more heavily on information about effectiveness of a drug. A similar pattern was found for pharmaceuticals used in agriculture. Knowing about these demographic differences may help to target communication materials to specific subgroups, such as younger adults and/or males.

Besides sociodemographic variables, it is possible that other interindividual factors might account for differences in risk perception and choices. One of these is the respondents’ health status. A recent study suggests that people with a poor health status would rather choose branded drugs instead of generic drugs for severe ailments [23], likely because they believe that only the best or priciest treatment is appropriate for their health. It is, therefore, likely that people with a poorer health status would be less willing to accept a drug with lower effectiveness. Inclusion of this factor may explain further variance in trade-off-decisions and in drug regulation acceptance.

Related, we expect that some of the respondents in this study will have been better informed, and more knowledgeable, about the deleterious environmental effects of pharmaceutical products than others. It is reasonable to hypothesize that these more knowledgeable people would have been more careful about their decisions to use pharmaceuticals, especially when they were accompanied by negative environmental consequences. Because we survey a random sample of adults, we are confident in the general findings drawn from this study; namely that the environmental impact of a drug is discounted in decisions about treating severe ailments. However, in a future study, it would be interesting to explore interactions between risk perception, decisions, knowledge, and—as alluded to above—respondents’ health status.

It is important to note also that our study only surveyed people living in the United States. Risk regulation of pharmaceuticals as well as handling of drugs is likely to differ between various countries. Furthermore, in contrast to many other countries, it is possible in the U.S. to buy pharmaceuticals not only in pharmacies but also in locations such as supermarkets and gas stations. Hence, U.S. residents should be quite familiar with evaluating different drug options that are available to them. Thus, it is not clear whether the results from this study can be transferred one-to-one to other countries.

## Conclusions

Based on the results of the present study, it can be assumed that the environmental impact of a drug will not play a large role in decisions about pharmaceuticals for severe diseases like cancer, in part because cancer is a highly dreaded disease. However, for less severe health risks, our results suggest that people are willing to forego some benefits associated with pharmaceutical products when the health of the environment is at risk. This willingness to accept a less effective drug in return for fewer negative environmental impacts was also observed for drugs used in agricultural production, where the benefits to humans are only indirect (if known at all).

Overall, our results also suggest that older adults and females are more aware of, and willing to act on, the environmental consequences of human and agricultural pharmaceuticals. These results have implications for the communication of the risks and benefits of drug use, handling, and disposal in both private household and agricultural settings.

We suggest that a future study could examine the role of price in people’s willingness to accept a less effective OTC drug for the benefit of the health of the environment. In addition, and at least for drugs available over the counter, it is possible that a label on environmental impact of a drug will influence people’s decision. Thus, a subsequent study could also examine the acceptance and effectiveness of such labels on OTC drugs. As a prerequisite, however, information on the environmental impact of a drug should be both distinct and visible.

## Consent

This research was approved by, and conducted in accordance with the policies of, the Institutional Review Board at Michigan State University (East Lansing, MI). Informed consent was obtained from each participant prior to their involvement in the study.

## Abbreviations

KN: Knowledge networks; OTC: Over the counter; US: United States of America.

## Competing interests

The authors declare that they have no competing interests.

## Authors’ contributions

SD conducted the data analysis and prepared the manuscript. VC designed the study, has been involved in drafting the manuscript and revising it critically for important intellectual content. JA designed the study, has been involved in drafting the manuscript and revising it critically for important intellectual content. All authors read and approved the final manuscript.
